# Addressing the challenges in projects of water treatment plants and storage of potable water: a case studies of the water supply system in the state of Goiás, Brazil

**DOI:** 10.1007/s42452-021-04382-1

**Published:** 2021-02-27

**Authors:** Anne Louise de Melo Dores, Felipe Corrêa Veloso dos Santos

**Affiliations:** Department of Engineering, Pontifical Catholic University of Goiás, Goiânia, Goiás Brazil

**Keywords:** Water systems efficiency, Water quality, Volume, Capacity, Consumption variation curve

## Abstract

To elaborate efficient and economical water supply systems is one of the main objectives in the sanitation companies water system projects. In order to address the challenges faced in reaching this objective, this study aims to identify, first, the relation between the percentage of non-conformed samples in treated water and the inefficiency of the filtering units installed in the water treatment plant, and second, if, by drawing the consumption variation curve it is the most efficient way to predict the storage tanks volume—comparing necessary capacity, determined by the consumption curve, and installed capacity, predict by the outdated Brazilian normative. In order to reach answers for these two questions, this study measured the operating efficiency of the treatment plant as well as have set a quantitative comparison between the two dimensioning criteria for storage tanks volume present in the literature. As a result, the analysis provided the authors to detect a focus of contamination in the single-layered filtering units, limited by the filtering capacity of 2–6 m^3^/(m^2^ day), whilst operating at 333.13 m^3^/(m^2^ day). As well as to detect by the drawing of the consumption variation curve an oversize of 68% and 60% in the dimensioning of the studied storage tanks. With the results provided by this analysis approach, it was possible to efficiently detect and correct critical impairments in the treatment phase and to conclude that a long-term analysis should be drawn in order to affirm if the consumption variation curve is the best design methodology for the reservoirs.

## Introduction

In the year of 2018, the Brazilian survey data SNIS [1] estimated a percentage of 83.62% of the country’s total population with access to treated water, corresponding to almost 35 million Brazilians without access to this basic service. Of this percentage, 14.3% are children and adolescents who do not have access to water, with 6.8% of these children and adolescents without a water system inside their homes [2]. Considering the demographic data for the same year of 2018, the state of Goiás (Brazil) reached a supply rate of 96.9% [3].

In the last 10 years, the state of Goiás grew above the national average, attracting people to new opportunities, mainly to the capital and metropolitan region. Despite the concentration of the population in large urban centres being still higher than in the interior, several cities in the interior of the state have been undergoing a process of exponential population growth in recent decades. This growth is mostly due to the migratory flow of people directly encouraged by the construction of highways and the intense agricultural activity [4], affecting the city's sanitary arrangement, especially in relation to water supply.

Despite an increase in population, a quarter of the municipalities in Goiás have lost their population, revealing that the growth is not homogeneous, that is, of the 246 municipalities in the State, 64 had a decrease in the population. Where the reverse happens, in the case of a disorderly growth, it makes it difficult for local governments to plan and provision basic services, such as sanitation [5].

Due to the important connection between the efficient treatment of water for distribution and the needs related to population health, the water supply system components must pass through a thorough analysis, in order to accurately detect impairments and improve the system, maintaining the efficacy in terms of quality and quantity, as well as being a form of reduction of implementation costs when onerous.

In terms of quality, the expansion and population growth of the interior cities directly affects the sanitary arrangement project by the Sanitation Company. It is the case of the city of Nova Crixás (Goiás, Brazil), where an alteration in non-conformed samples is possibly related to a focus of contamination in the treatment units, provoked by the increase in demand. The population expanded and the treatment units remained the same.

Conventional water treatment plants, according to the literature [6], have limits of operational capacity. The flocculator operates at a detention time ranging from 20 to 30 min, the decanter with a runoff rate limited by 25 m^3^/(m^2^ day)—for treatment plants with capacity ranging between 1.000 and 10.000 m^3^/day, and slow sand filtering units with filtration rate between 2 to 6 m^3^/(m^2^ day) [7].

Under appropriated circumstances slow sand filters can be the simplest and cost-effective way of treatment, with some peculiarities implied that prevents its wider use [8]. There is a limited range of suitable water quality, as generally there is no chemical pre-treatment, and is not recommended to apply residual oxidant such as chloride, previously to filtration. It is important to maintain a slow and constant filtration rate, whereas this is the responsible for the development of the biological process of the filter, a thin layer denominated *schmutzdecke* [9], responsible for contributing to the removal of turbid particles of the water.

When these peculiarities are not specifically met, there is a possibility of the existence of non-conformity samples in water quality index be related with the impairment of the slow sand filtering units, especially when installed in treatment plants of countryside cities that have undergone a process of populational growth driven by economic activity.

Regarding the economical aspect, it is important to take into consideration that sanitation is an area that encompasses a large part of government investments, especially for developing countries. In Brazil, during the years of 1971 and 1986, the implementation of the PLANASA, a sanitation incentive project, the government invested the equivalent to R$ 8.5 billion per year during the sixteen years of the project. From the end of PLANASA to the year of 2006, the investments in the sector decreased by no more than R$ 4,5 billion per year, until the Law 11.445 of 2007 that tripled the investments in the sector by the year of 2017. However, despite these expressive values, the vast majority of water and sewage company operators are under economic and financial fragility [10].

As the implement of storage tanks consists big part of the investment of the water systems, studying new ways of minorize these implementation costs are a benefit. In the state of Goiás, the sanitation company, due to the lack of data reliability, do not use the consumption variation curve in the design of the water supply system project, adopting the criterion of the old normative for the volume of the storage tanks and generating inaccuracy between reservation volume and actual demand.

Therefore, the hypothesis of this work is to address these two challenges faced by the sanitation company of the state of Goiás (Brazil), combining analysis in demand, water quality, and treatment capacity. First, by proving the relation between the existence of non-conformity samples in water quality index and the impairment of slow sand filtering units, when analysing the operational capacity of the treatment plant. And second, by testing the efficiency and functionality of the use of the consumption curve to predict the storage tanks volume, comparing it with the volume installed in the system today.

### Objectives

1. Relate the percentage of non-conformed samples in treated water with the inefficiency of the filtering units installed.
2. Draw the consumption variation curve in order to measure the efficiency and functionality of its use in the design of the water tank system.

## Methodology

### Water Treatment Plant: potability standard

**Fig. 1:**
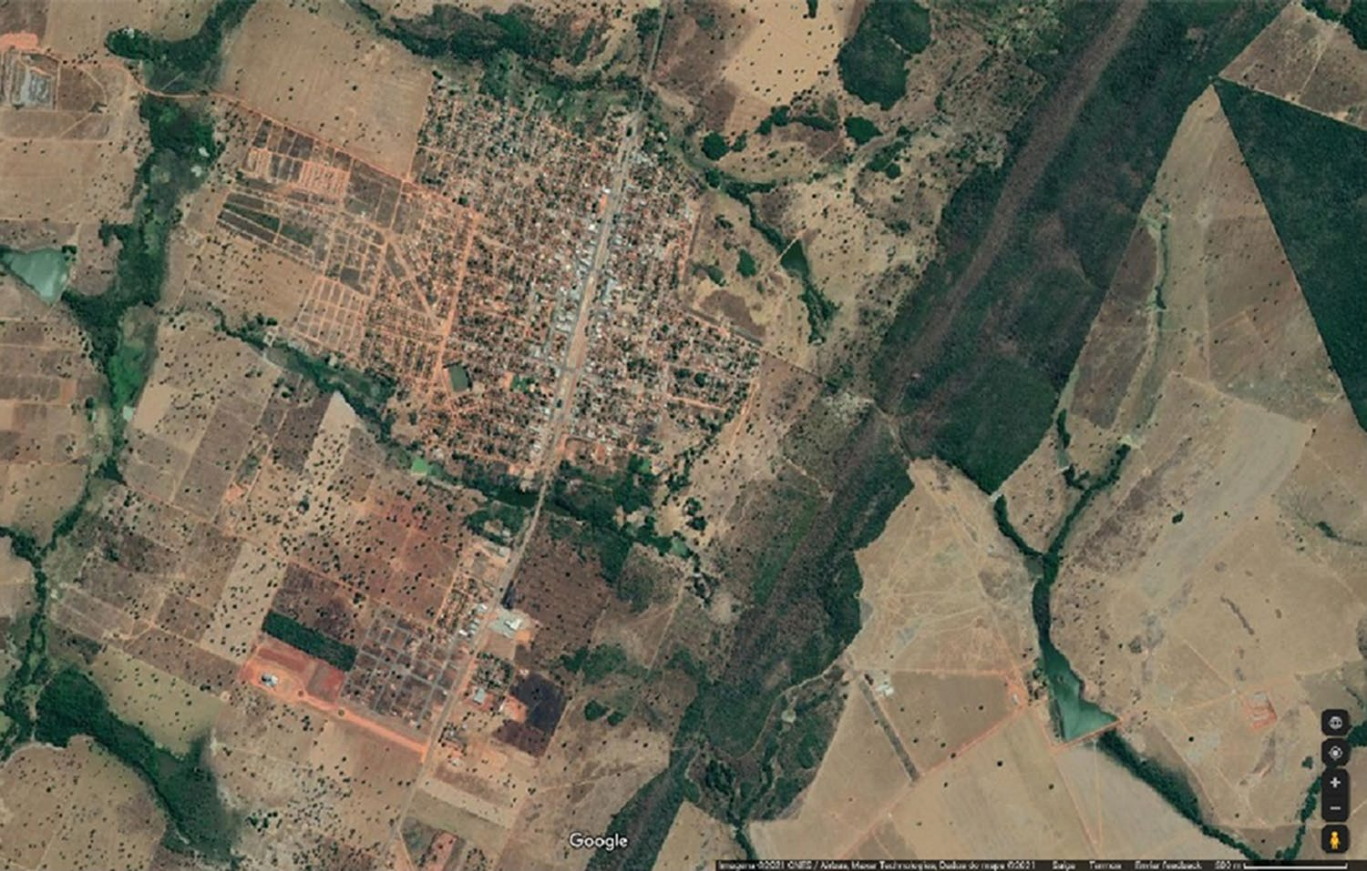
District of Nova Crixás, Goiás, Brazil

In this study was analyse the operating conditions of the water treatment plant of the city of Nova Crixás, located in the state of Goiás (Brazil) and operated by the state sanitation company (Fig. 1)

Nova Crixás is today the 5th largest municipality in territorial extension of the state and is the first as holder of the largest cattle herd in the state, the 12° in the country. Taken to the category of municipality and district on 1 February 1982 has a population density of approximately 1.8 inhabitants/km^2^.

**Fig. 2:**
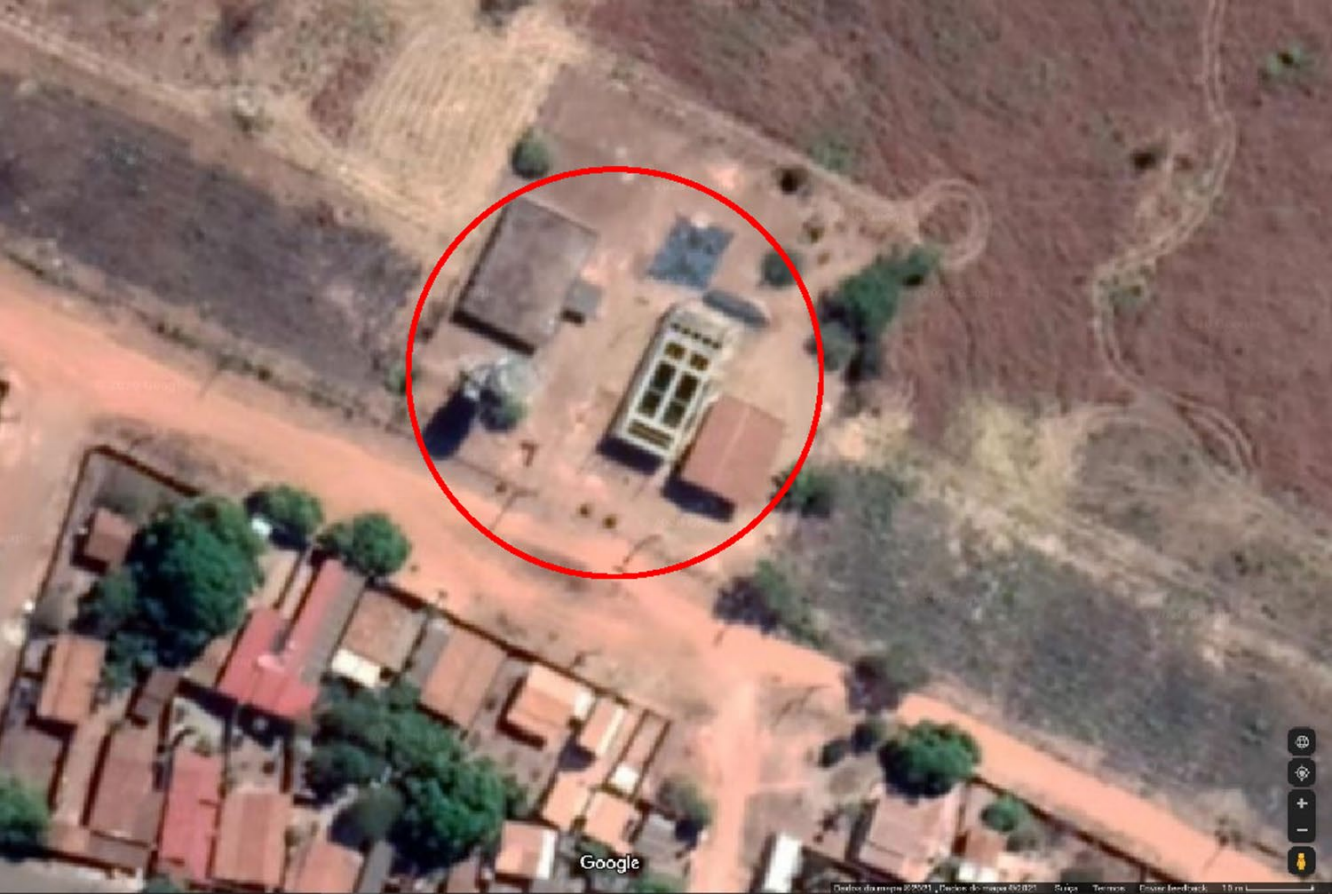
General view of the location of the water treatment plant and treatment units

Figure 2 presents the disposition of the treating units in the water treatment plant. The raw water of the system is pumped from the ‘Brejão’ Creek by the suction well and transported to the treatment plant where the conventional treatment process happens. The water pumped from the creek goes to the entrance chamber, where will be directed to the two flocculator units, Alabama type, and consequently sent to the other two decanters, conventional type, that feeds all the five slow sand filtering units of the system. When treated, the water fills two water tanks, one elevated and one in the surface, located on the extreme left of the precinct in Fig. 2.

Using the company’s operational data system, monthly water flow produced by the Treatment Plant in a period of one year was collected in order to determinate the average and maximum capacities of treatment, as illustrated in Table 1.

**Table 1:** Water flow produced in the water treatment plant. Data provided by the operating system OP030 (2018), of the sanitation company SANEAGO

Month/Year	07/2017	08/2017	09/2017	10/2017	11/2017	12/2017	–
Water flow (L/s)	30.61	30.56	26.54	15.11	20.46	24.10	–
Operation (hours/month)	480.34	488.17	578.56	694.55	487.45	434.27	–

Month/Year	01/2018	02/2018	03/2018	04/2018	05/2018	06/2018	07/2018
Water flow (L/s)	21.69	26.58	30.68	27.67	23.41	21.14	20.71
Operation (hours/month)	489.38	415.16	446.04	475.54	591.00	528.11	567.57
Maximum water flow (L/s)	31						
Average water flow (L/s)	24						

The data for maximum and average water flow were rounded up merely for the purpose of calculation and do not interfere in the outcome result of this analysis

To analyse the installed capacity of the treatment components in this study, the authors considerate the maximum water flow rate of 31 L/s.

As the system produced water with a Water Quality Index of 97.04% a percentage of non-conformed samples were detected for substances as total coliform, colour, and turbidity that are parameters of analysis recommend by the Ministry of Health [11]. Tables 2 and 3 illustrate the results of non-conformities. In order to detect the cause of the impairment, all the treatment units—flocculate, decanter, and filtering units, were analysed in terms of operational capacity.

**Table 2:** Irregularities in the Water Quality Index performed in the treatment station. Data provided by the operating system of the laboratory of water quality LQA083 (2018), of the sanitation company SANEAGO

Irregularities	Water treatment station										
Analysis	5—TURB	6—COLOUR	7—pH	4—F	3—Cl	11—Fe	12—Al	28—Mn	20—Total Coliform	21—*E.coli*	Total
% Non-conformity	2.38	1.75	0.00	2.97	1.78	0.00	5.00	0.00	0.59	0.00	1.38

**Table 3:** Irregularities in the Water Quality Index performed in the outlet of the treatment station. Data provided by the operating system of the laboratory of water quality LQA083 (2018), of the sanitation company SANEAGO

Irregularities	Outlet										
Analysis	5—TURB	6—COLOUR	7—pH	4—F	3—Cl	11—Fe	12—Al	28—Mn	20—Total Coliform	21—*E.coli*	Total
% Non-conformity	3.38	3.42	0.00	0.00	3.38	0.00	0.00	4.00	0.56	0.00	2.05

#### Flocculation and detention time

In operation at the conventional water treatment plant of Nova Crixás, two hydraulic Alabama-type flocculation units are responsible to promote the aggregation of the particles formed at the previous stage of treatment. The capacity of these units is expressed by the time of detention of the particles, sufficient for the biological reactions to occur. The units installed at the treatment plant works satisfactory with levels for detention time [6] ranging from the following:

$$20\;{\text{minutes }} \le {\text{ Detention Time }} \le \, 30\;{\text{minutes}}$$

To determine the capacity of the flocculate units installed in the treatment plant, the maximum treatment capacity was taken into consideration, in order to predict the worse-case scenario in which the units operate. Therefore, using Eqs. (1) and (2) with the project specifications from Table 4, the detention time for the Nova Crixás water treatment plant is determinate in Eq. (3).

**Table 4:** Flocculation units project specifications

Units	Base (m)	Length (m)	Depth (m)	Water Flow ‘Q’ (m^3^/s)
2	1.25	8.05	2.85	0.031

As the authors already have the project specification, the superficial area ‘As’ is determined by Eq. (1) regarding the calculus of superficial area for rectangle section in floor plan.

1 $${\text{As}} = 2 \times b \times l \left( {{\text{m}}} \right)$$

Volume in Eq. (2) calculated between superficial area and depth.

2 $${\text{Volume}} = {\text{As}}\, \times \,{\text{depth}} \left( {{\text{m}}^{3} } \right)$$

Detention time for two flocculation units in operation in Eq. (3), according to the literature [12].

3 $$t = \frac{{\text{volume capacity}}}{Q} \left( {\text{s}} \right)$$

#### Decanter and runoff rate

Runoff rate is the parameter adopt to characterize the Decanter Efficiency [12]. According to the item 5.10.2 of the Brazilian normative [6], when laboratory tests are not able of being proceed, the runoff rate of a conventional water treatment plant with capacity ranging between 1.000 and 10.000 m^3^/day is limited by 25 m^3^/(m^2^ day) in order to guarantee a good operational control.

Determinate by the relation between the treatment flow rate and the surface area of the decanter unit, runoff rate is estimate in Eq. (4), according to the literature [6].

4 $${\text{Runoff}} {\text{Rate}} = \frac{Q}{{{\text{As}}}} \left( {{\text{m}}^{3} /\left( {{\text{m}}^{2} \times {\text{day}}} \right)} \right)$$

For transforming the water flow rate ‘Q’ from L/s to m^3^/day, was taken in consideration the operation time of 488.17 h/month from Table 1, correspondent to the maximum capacity of 31 L/s and transformed to hours/day in Eq. (5).

5 $$M = 488.17 \frac{{{\text{hours}}}}{{{\text{month}}}} \times \frac{{\text{1 month}}}{{\text{30 days}}}$$

$$M = 16.27 \;{\text{h}}/{\text{day}}$$

Therefore, the water flow rate used in Eq. (4) is calculated by Eq. (6).

6 $$Q = M \times 3600 \times 31 \times 10^{ - 3}$$

$$Q = 1815.73 \left( {{\text{m}}^{3} /{\text{day}}} \right)$$

The project dimension of the decanter units used for the calculus of Superficial Area (1) are in Table 5 bellow.

**Table 5:** Decanter units project specifications

Units	Base (m)	Length (m)
2	3.90	11.00

#### Filtering units and filtration rate

Important components of the system, filters have a large particle retention capacity. The five filtering units of the treatment plant in Nova Crixás, Goiás, covering each an area of 8.04m^2^, works with a single layer of filtering material. According to the literature [7], the expected filtration rate for single layered filters should be ranging from 2 to 6 m^3^/(m^2^ day), expressed by Eq. (7) as the reason between water flow rate and the filtering unit area [13].

7 $$q = \frac{Q}{A} \left( {m^{3} /\left( {m^{2} \times {\text{day}}} \right)} \right)$$

The capacity limit of this filters is exceptionally low, therefore used in small communities only. When in case of impairment, with the aim of maintaining the quality of the water without altering the dimensions projected for the filters, the replacement of the slow filtration filters by a high-rate filters with a double layer of Sand + Anthracite and upward flow, is recommended as a cost-effective solution [14]. High-rate filters can support the actual filtration rate, relieving considerably the filter overload with a limit of 360 m^3^/ (m^2^.day) [6].

Following the determinations of the normative [6] for High-Rate filters, by Eq. (8) was possible to estimate the amount of material for the two layers of sand and for the one of anthracite, for each filtering unit.

8 $${\text{Volume}} = {\text{Filter}} {\text{Base Area}} \, \times \,{\text{Layer}} \;{\text{Thickness}} \left( {{\text{m}}^{3} } \right)$$

For high up flow rate filters, to be implement on existing filters the layer of anthracite must have a thickness of 0,50 m and the layer of sand must have a thickness of 0.40 m (divided into 0.20 m of thin sand and 0.20 m of thick sand) with granulometry of the sand being, respectively, 0.5 mm and 0.8 mm. In order to comply with the normative [6], the filtering layer plus the backing layer must necessarily be at least 40 cm below the pipe gutter, agreeing to the 30% expansion of the layer provided by the norm.

When applying Eq. (8) to estimate the volume of material for the 0,50 m anthracite layer, Eqs. (9) and (10) estimates the quantity of 25 kg sacs of anthracite necessary for each filtering unit:

9 $${\text{mass}} = {\text{anthracite density}}\, \times \,{\text{volume}} \left( {{\text{kg}}} \right)$$

10 $${\text{quantity}} {\text{of}} 25 {\text{kg}} {\text{sacs}} = \frac{{{\text{mass}}}}{25}$$

### Potable water storage

**Fig. 3:**
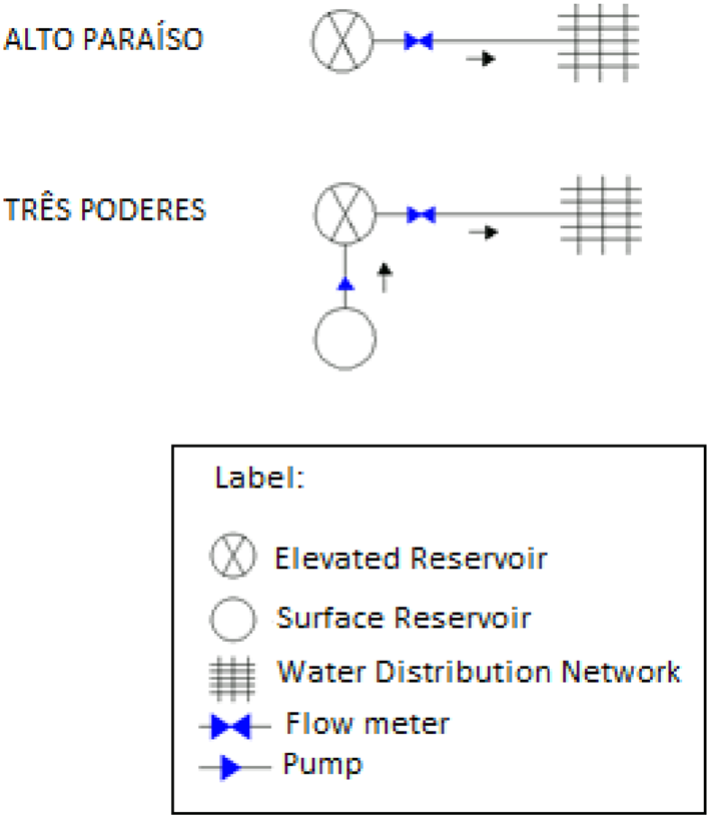
Schematic of the Itaguari Service Reservoir System

As a reference in system automation in the state of Goiás, Brazil, the city of Itaguari presents expressive amount of data and a considerable reliability when in terms of water flow measurement in the service reservoirs. The city is supplied by one elevated reservoir denominated Alto Paraíso of 50 m^3^ and one elevated reservoir named Três Poderes of 200 m^3^ complemented by a surface unit of 150 m^3^, as demonstrated in Fig. 3.

The data provided by the sanitation company, compiles flow rate measurements of every fifteen minutes in a period of one year from September of 2018 to August 2019. In order to generate the consumption variation curve, the data were filter into an average flow rate per day of higher consume in the month, for each elevated reservoir, making possible to detect the day with highest water consumption in the period. Table 6 and Table 7 demonstrate the filtered data. From this analysis is determinate the day with the maximum consumption for the period, of which will be the basis of the volume capacity calculus.

**Table 6:** Reservoir Três Poderes: Average consume per day

Reservoir Três Poderes	Average flow rate (m^3^/h)
07/09/2018	32.68
12/10/2018	31.96
15/11/2018	27.85
22/12/2018	30.78
03/01/2019	31.31
02/02/2019	31.83
12/03/2019	36.95
12/04/2019	28.67
10/05/2019	31.04
21/06/2019	32.17
12/07/2019	29.23
16/08/2019	31.20
Maximum average consumption	36.95

**Table 7:** Reservoir Alto Paraíso: Average consume rate per day

Reservoir Alto Paraíso	Average flow rate (m^3^/h)
07/09/2018	3.65
12/10/2018	3.44
16/11/2018	2.94
22/12/2018	3.36
18/01/2019	3.67
02/02/2019	3.29
16/03/2019	3.26
27/04/2019	3.40
11/05/2019	3.31
15/06/2019	3.28
13/07/2019	3.24
17/08/2019	3.53
Maximum average consumption	3.67

#### Consumption variation curve

The consumption variation curve is the method chosen to define the utile volume of the reservoirs that attend the variations of water consumption, considering continuous abduction of water, in a period of 24 h of the day with the highest consume of water.

Having as a reference the day of highest consumption of water, it was set a percentage of variation in the order of 4% of the highest flow rate, to generate other days of the month consumption curve, and consequently detect eventual inconsistence in the data provided by the sanitation company.

#### Dimensioning of the reservoirs

The dimensioning of the elevated reservoirs was calculated through Eq. (11) based on the consumption variation curve as recommended by the current Brazilian normative [15], which sets the requisites for the project of reservoirs for public supply.

11 $$V = {\text{ }}\int_{{t_{1} }}^{{t_{2} }} {Q{\text{dt}} - Q_{{{\text{med}}}} \left( {t_{2} - t_{1} } \right)\left( {{\text{m}}^{3} } \right)}$$

where *Q* is the water flow consumed at the day, *Q*_med_ the average of water consume, *t*_2_ the instant of which the water consume is lower than the water flow supply and *t*_1_ the instant of which the water consume is higher than the water flow supply.

The current normative [15] recommends applying the factor *k*_1_ = 1.2 to the volume calculated to consider the uncertainties of the data used. Also, the normative provides that in the absence of reliable data, specific technical–economic study should be carried out to justify the value adopted.

Nowadays, the sanitary company of the state of Goiás use the recommendations of the outdated normative [16] in the dimensioning of the reservoirs for the water supply system. In the absence of data for the variation curve of consumption, it is recommended to consider continuous water adduction (24 h) for the storage volume greater than or equal to one third of the volume distributed on the maximum consumption day. Therefore, the dimensioning of the storage tanks is demonstrated by Eqs. (12) and (13) [16] in order to compare the results of volume capacity between the two normative.

12 $$Q_{{{\text{med}}}} = \frac{{n \, \times \,{\text{ocupation rate}}\, \times \,q \times k_{1} }}{86.400} \left( {\text{L/s}} \right)$$

where ‘*n*’ stands for the number of houses with potable water, ‘*q’* the per capita consume adopted by the sanitation company as 150 m^3^/m^2^ day and *k*_1_ as the daily variation coefficient as 1.2.

13 $$V = \frac{1}{3} \times Q_{{{\text{med}}}} \left( {{\text{m}}^{3} } \right)$$

## Results and discussions

### Water Treatment Plant diagnosis

The main source of water caption in the state of Goiás, Brazil, is through surface water. Those commonly does not express potability quality, demanding treatment before being ready for public consume. Therefore, the Water Quality Index is one of the most important qualitative potability standards, setting acceptable levels for a minimum of quality for water to public consummation.

Tables 2 and 3 demonstrate the percentage for each nonconforming analysis obtained through the operating system of the laboratory of water quality (LQA083-2018). These values in juxtaposition with the diagnosis of the filtration unit in Table 8 correlate in cause and effect, as a low filtration rate under extreme operating conditions led to the decrease of the water quality. The filtration rate calculated shows that under the current conditions the filter is by far overloaded and therefore does not fit the standards recommended by the Ministry of Health [11] for potable water.

**Table 8:** Diagnosis of the operating conditions of the treatment units

Treatment unit	Limit capacity^a^	Operating level	Diagnosis
Flocculation	20–30 min^1^	31 min	The unit operates above its limit capacity
Decanter	25 m^3^/(m^2^ day)^b^	21.16 m^3^/(m^2^ day)	The unit operates within its limit capacity
Filter	2–6 m^3^/(m^2^ day)^c^	333.13 m^3^/(m^2^ day)^b^	The unit operates well above its limit capacity

^a^Limit capacities predict in the literature

^b^Brazilian Association of Technical Normative ABNT NBR 12,216 (1992)

^c^Seckler SF (2017)

Slow sand filters are suitable for small communities and therefore small systems [8]. In the principle, Nova Crixás was adapted to this concept and therefore did not demanded a more sophisticated system. With the development of the region, the increasing demand, driven by the city expansion, led to the impairment of the filtering units compromising its service life.

Alongside with the low filtering capacity, another disadvantage of the slow sand filters is that, in order to broaden the range of source water that can be successfully treated in the units, it is recommend [8] to combine pre-ozonation or roughing filters in the treatment.

Although the two Alabama-type flocculate units are not working within its theoretical limits, even if remarkably close to the correct parameter, this diagnosis, compared with the filter performance, exempt the flocculate unit as the main cause of contamination of the treatment system. However, there is a possibility that the units work with a different hydrodynamic behaviour, hiding hydraulic events that leads to a ‘real detention time’ different from the theoretical one [17].

As the decanter unit operates within its limit capacity, no major concerns were raised. Moreover, under the present conditions, demands were not an intervention.

Due to the development and population growth of Brazilian cities, the diagnosis of operational and technical activities is of utmost importance for the improvement of the water quality and consequent adaptation of the component units of the Water Treatment Stations. Those responsible for the system operation, in this case the State Sanitation Company, SANEAGO, are able to report all non-conformities to the Municipal Health Secretariat and to make the necessary changes to ensure good water quality [18].

As a cost-effective solution to the filtering unit impairment, the replacement of the Slow Filtration Filters by a Rapid Filter, with a double layer of Sand + Anthracite, maintains the dimensions of the Filter in design and the same flow rate, relieving the filter overload. The new filtering limit capacity is raised to the equivalent of 360 m^3^/(m^2^ day) [6], guaranteeing an extended service life to this units and assuring quality in the water treated in the filters. Table 9 determinates all the volume of materials necessary for the five filters unit adaptation.

**Table 9:** Volume of materials necessary to adapt five filtering units

Filtering material	Volume (m^3^)	Quantity (per bag of 25 kg)
Thick sand—0.8 mm	8.05	–
Thin sand—0.5 mm	8.05	–
Anthracite	20.01	885

**Fig. 4:**
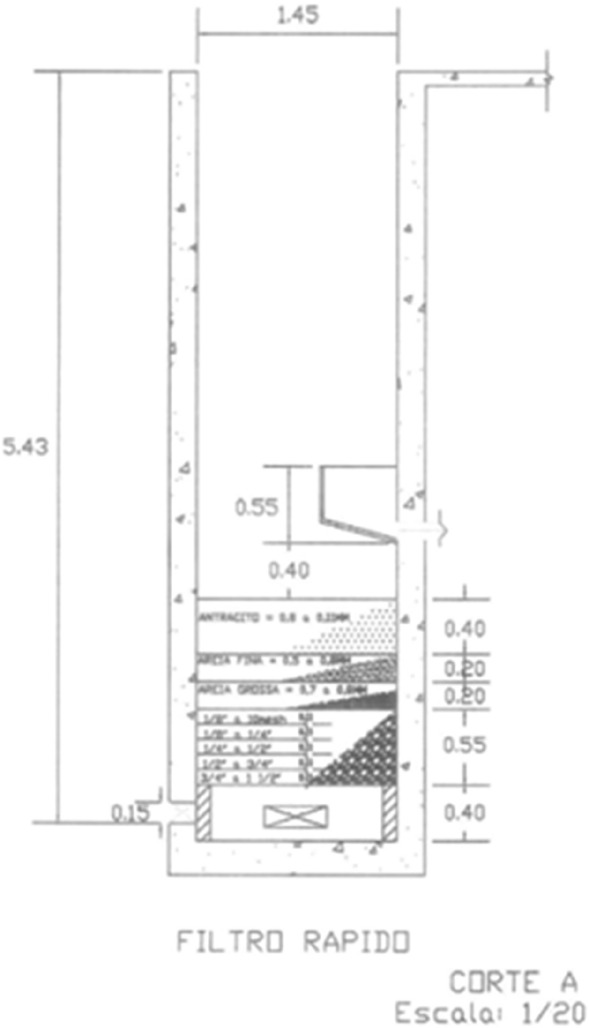
Conceptual subdivision of the rapid rate filter

Figure 4 illustrates the proposition for each filter, working as high-rate filters with a double layer of sand and a single layer of anthracite.

By changing to a High Filtration Rate as proposed, the filtering unit capacity expands to 33 L/s and the efficiency of the treatment system will be within the theoretical and normative limits present in the literature. However, this adaptation does not exempt the need for expansion of the water treatment plant. Although it is a cost-effective solution, in a long-term, in case no future capacity expansions are made, the system will continue to be in disability according to the parameters established by the Ministry of Health.

### Storage of potable water

In reference to the Storage of Potable water, from the data generated for the average flows of each month of Tables 6 and 7, it was possible to make a graphical analysis of the system implemented in the city of Itaguari for each reservoir.

**Fig. 5:**
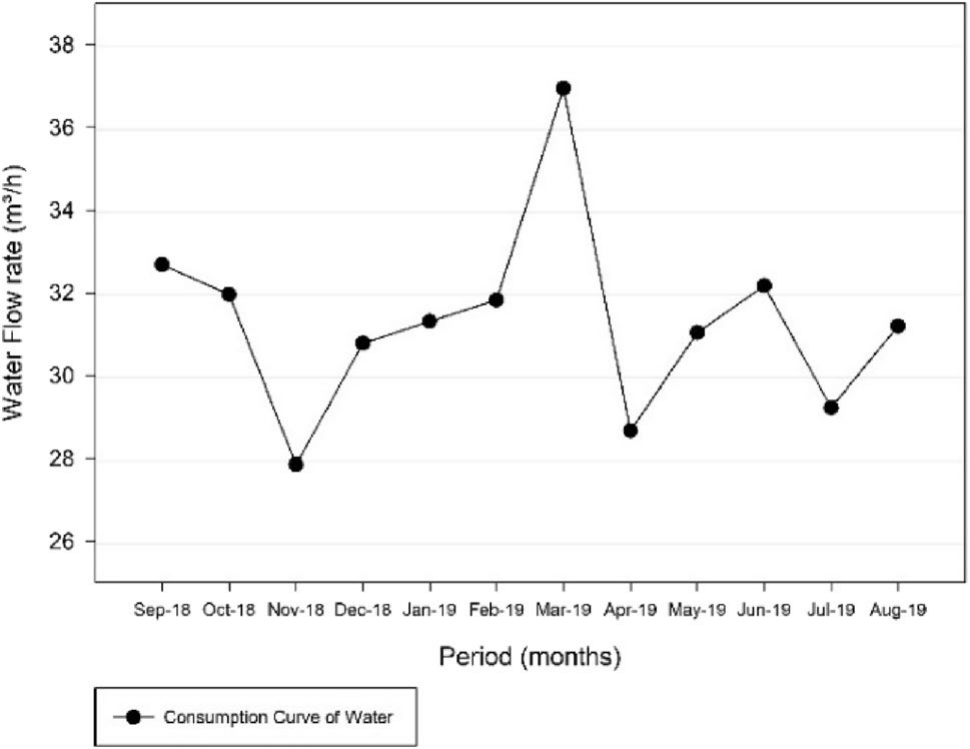
Consumption Curve of the Três Poderes Elevated Reservoir, for the average flow rate of each month between September 2018 and August 2019

Regarding the Três Poderes Elevated Reservoir, a consumption curve for the average daily flows between September 2018 and August 2019 is represented by Fig. 5.

**Fig. 6:**
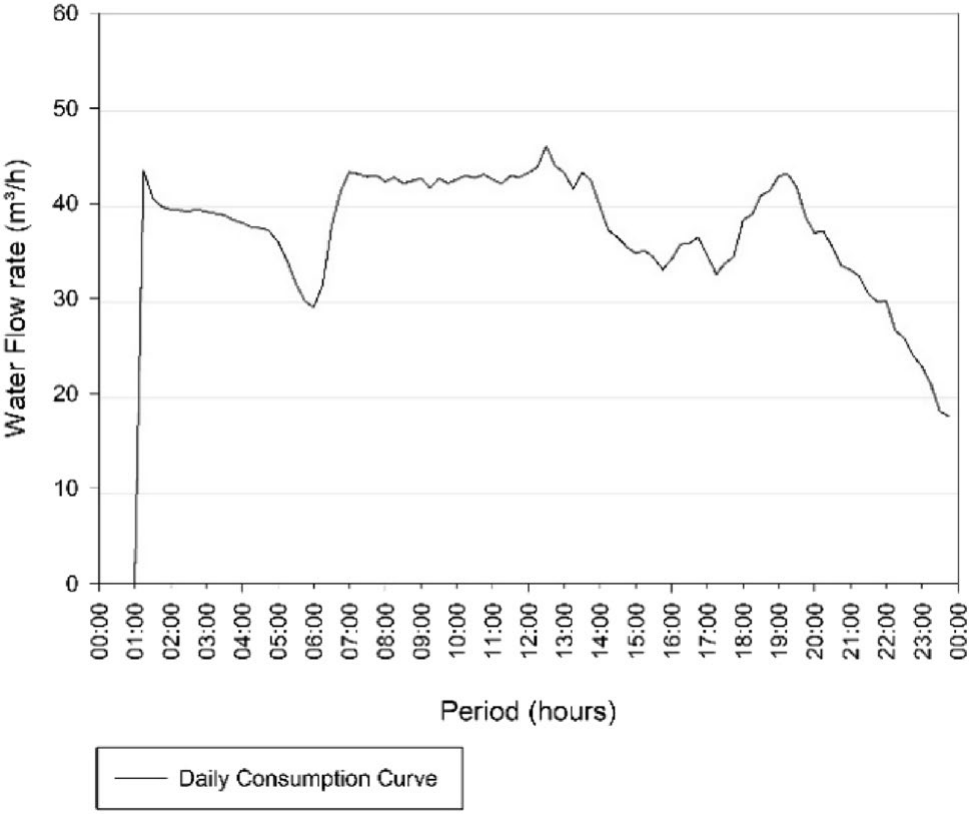
Consumption Curve of the Três Poderes Elevated Reservoir for the day of highest consumption on March 12 of 2019

Figure 5 illustrates that the day of highest consumption is in March 2019, corresponding to March 12 of 2019 and an average flow of 36.95 m^3^/h. With respect to the other values, that day is represented in the graph at a point very distant from the other values that vary from around 30–35 m^3^/h. Therefore, the consumption curve for that day was generated in order to diagnose this discrepant flow variation, as shown in Fig. 6.

When analysing the consumption curve for the hottest day of the year, referring to March 12 of 2019, an inconsistency in the data was detect. Figure 6 of the consumption curve does not match with the predict in literature [19] for the peak consumption for 24 h contained between 8 am and 7 pm. This because the values of consumption at dawn are remarkably close to the values during the peak period, making it impracticable to consider this day as the one with the highest consumption of the year and generate the volume from it (Fig. 6).

Therefore, the data for March 12 of 2019 were invalidated in this study because are not reliable. It was not possible to identify the reasons that led to these results since access to information and contacts with SANEAGO operators were more restricted due to the social isolation to which the country was subject due to COVID-19.

**Fig. 7:**
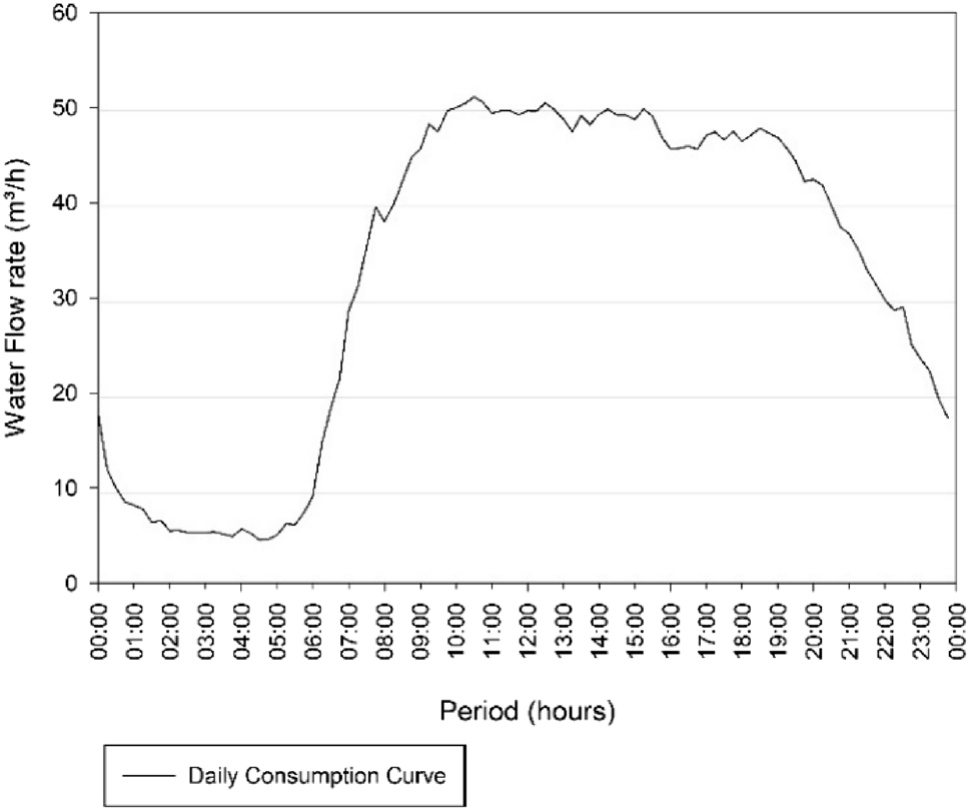
Consumption Curve of the Três Poderes Elevated Reservoir for the day of highest consumption on September 7 of 2018

Then, with a second analysis of Fig. 5, the day of highest consumption was September 7 of 2018, which presents the second highest average of daily flows, referring to 32.68 m^3^/h. The consumption curve corresponding to this day is demonstrated in Fig. 7.

In Fig. 7 the curve follows the pattern of the literature [19], where the highest consumption occurs in the peak period (8:00 am to 7:00 pm), while in the early morning the consumption is low (00:00 to 6:00 am), according with the habits of the population.

**Fig. 8:**
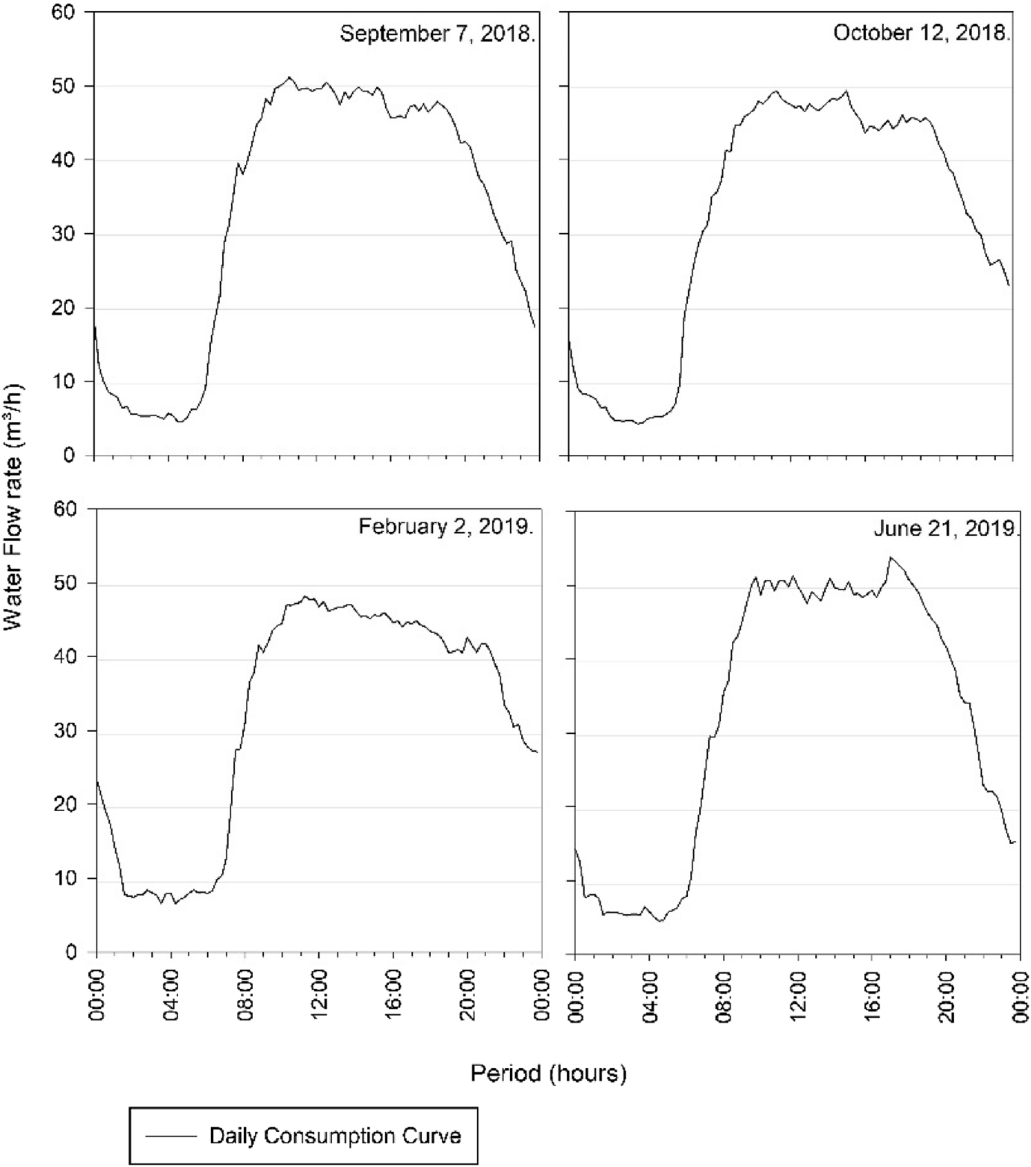
Três Poderes Consumption Curve for Highest Daily Flows between September 2018 and August 2019

The days that are within the variation of 4% of the day of highest consumption, and that were analysed to determine the volume of the Três Poderes elevated reservoir, are October 12 of 2018, February 2 of 2019 and June 21 of 2019. Therefore, four curves were study for the determination of reservation volume, according to Table 10 and Fig. 8.

**Table 10:** Average flows of the days of highest consumption for Três Poderes

Day of highest consumption	Average flow rate (m^3^/h)
September 7 of 2018	32.68
October 12 of 2018	31.96
February 2 of 2019	31.83
June 21 of 2019	32.17

From Fig. 8, the curves follow the pattern of the literature [19], where the highest consumption occurs during the day, while at dawn the consumption is lower, showing that there is a standard behaviour of consumers and that the data of March 12 need to be discarded in this study for presenting an atypical consumption for the city.

**Fig. 9:**
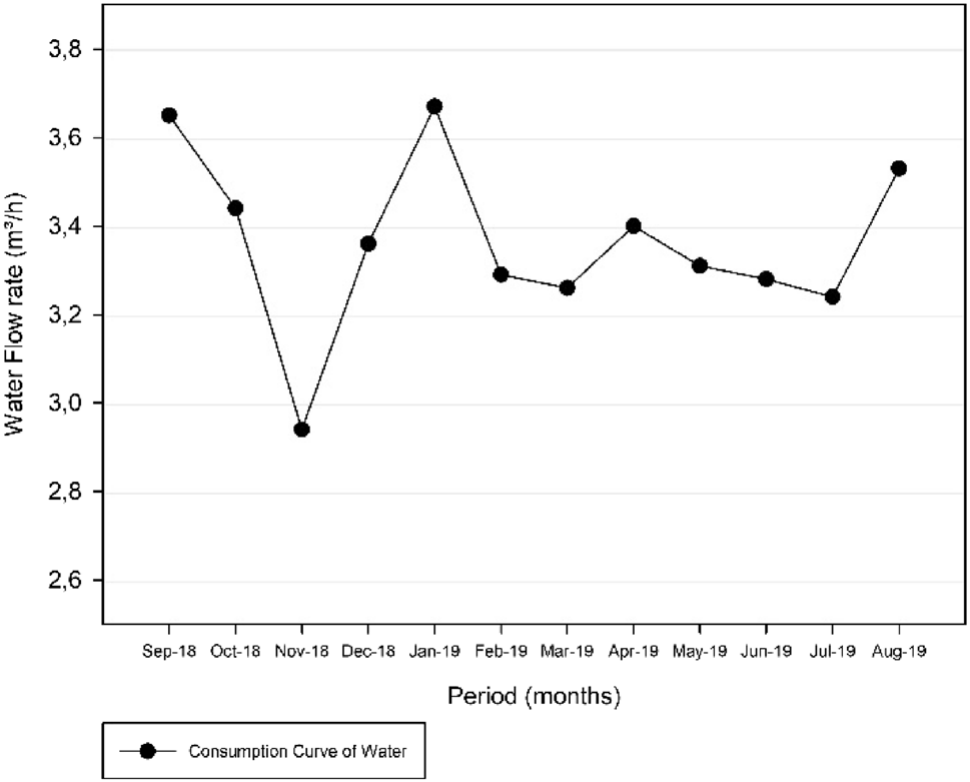
Consumption Curve of the Alto Paraíso Elevated Reservoir, for the average daily flow rate of each month between September 2018 and August 2019

Regarding the Alto Paraíso Elevated Reservoir, a consumption curve for the average daily flows between September 2018 and August 2019 is represented by Fig. 9.

By analysing Fig. 9, the day of highest consumption for the Alto Paraíso Elevated Reservoir is on January 18 of 2019 and corresponds to an average flow of 3.69 m^3^/h. This day is represented in the graph within the range of the other values, which vary mainly around 3.30–3.70 m^3^/h.

As there is no discrepant variation in the values between the day of highest consumption on January 18 of 2019 and the values for the other days, there is no evidence of inconsistency in the analysed data for this day, as occurred in the Três Poderes Elevated Reservoir. What is noted is a discrepancy in the data for November 16 of 2018, corresponding to an average daily flow of 2.94 m^3^/h. Nevertheless, as this day represents the lowest value on the chart, data for this day were not consider in this study.

**Fig. 10:**
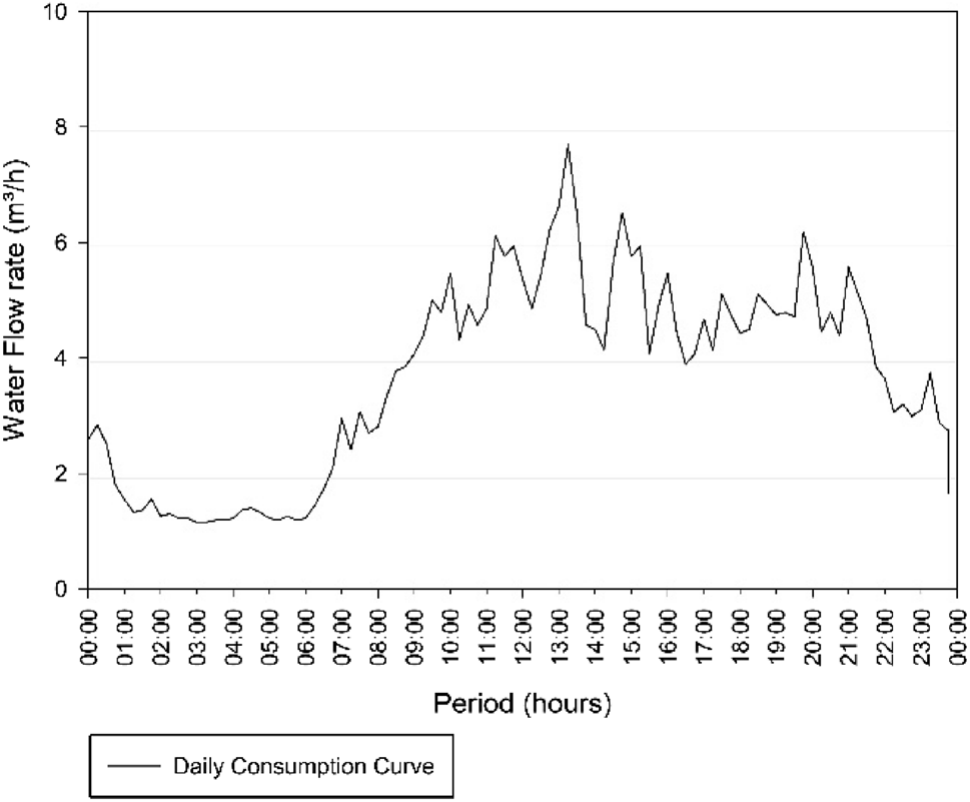
Consumption Curve of the Alto Paraíso Elevated Reservoir for the day of highest consumption on January 18 of 2019

Therefore, the consumption curve for the day of highest consumption of the year for this reservoir, corresponding to January 18 of 2019, is demonstrate in Fig. 10.

The graphical analysis of Fig. 10 allows confirmation of the viability of the data collected to determine the storage volume of the reservoir. The consumption curve meets the values presented in the literature [19], where the highest flow values are between 8 am and 7 pm with a peak between 12 and 1 pm.

**Fig. 11:**
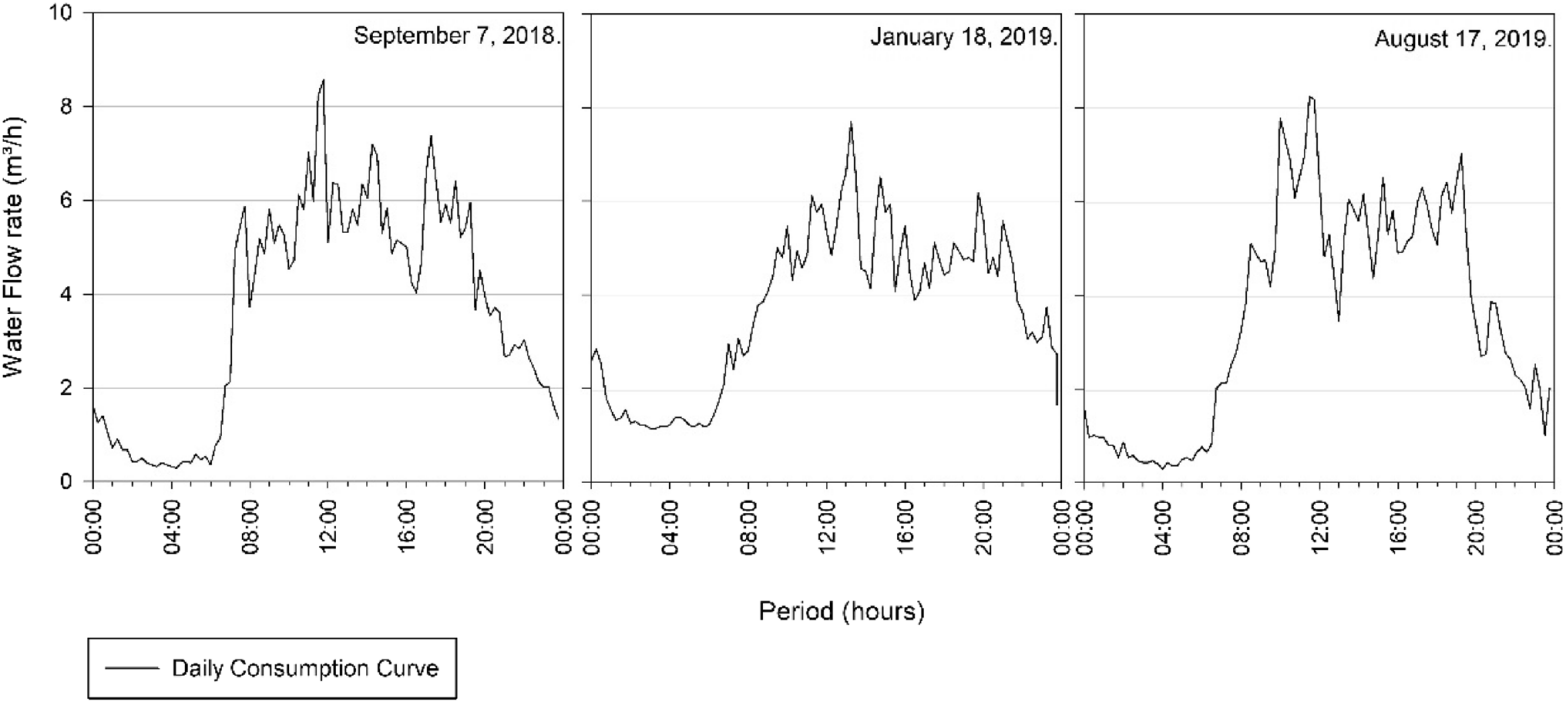
Consumption Curves for Highest Daily Flows between September 2018 and August 2019 for Alto Paraíso

The days with average flow with a variation less than the 4% adopted for determining the volume of the Alto Paraíso Elevated Reservoir are September 7 of 2018 and August 17 of 2019, according to data in the following Table 11 and Fig. 11, corresponding to the curves in study.

**Table 11:** Average flows of the days of highest consumption for Alto Paraíso

Day of highest consumption	Average flow rate (m^3^/h)
September 7 of 2018	3.65
January 18 of 2019	3.67
August 17 of 2019	3.53

Through the graphical analysis of consumption in Fig. 8 and Fig. 11, the curves of each system—Três Poderes and Alto Paraíso have different behaviours. For the Três Poderes reservoir, the consumption curves are smooth, while for the Alto Paraíso reservoir, the curves present many consumption peaks, characterizing a variation in demand during the day.

As Itaguari is relatively a small city, with a population of around 4676 inhabitants [20], it is not believed that this variation in consumption, shown in the graphics, is due to different habits of the population. What is common is that small towns have the same type of occupation, mostly houses, and the habits of the population are similar. However, it is believed that this variation in the behaviour of the curves is due to the flow measurement equipment itself.

Despite this difference, when analysing the behaviour of the consumption curves of each reservoir, for each hottest day throughout the year, these are similar to each other, demonstrating a coherent trend in the system's behaviour. This isolated analysis of the system makes it possible to detect inconsistencies in the consumption readings, as happened for the Três Poderes reservoir on March 12 of 2019, which was discard due to the atypical behaviour.

Another difference is that for the Três Poderes reservoir the day of highest consumption was in the month of September, while for the Alto Paraíso system the day of highest consumption was in January. September is one of the hottest months of the year in the state of Goiás, with a maximum temperature of 36° C, while January has a maximum of 31° C [21]. This explains the higher consumption to be relate to September for the Três Poderes system.

As for the Alto Paraíso system, as shown in Table 10 for average flow values, a difference of approximately 20 L/h separates the day of highest consumption on January 18 of 2019 and the second day of highest consumption on September 7 of 2018. These values are remarkably close and, when representing the January 7 curve, we use average flow values very close to the month of September, that is technically the hottest month in the state.

Given the above, it is possible to conclude that in order to guarantee a correct analysis of the consumption behaviour of the system it is of great importance to extend the analysis of consumption to other days than just the day of highest consumption of that year. In this way, it is possible to guarantee greater security in the choice of the day to be analysed, eliminating any unusual behaviour of the system.

When considering a macro aspect, for Sanitation companies this detailed analysis of the population's behaviour is currently unpracticable. This is because large investments have to be done aimed at operating the system, with sectorization of the network, installation of measuring equipment in the networks and outlets of reservoirs, technical courses for the system operators. Most municipal water supply systems do not have reliable data due to the factors mentioned [22]. Therefore, to analyse the population's consumption and calculate the volume, the Sanitation Company of the State makes use of a Standard Curve, used for the dimensioning of the reservoirs.

The volume of the reservoirs was then dimensioned according to both current [15] and outdated [16] Brazilian normative, for the day of highest consumption on September 7 of 2018, for the Reservoir Três Poderes and January 18 of 2019, for the Alto Paraíso Reservoir.

### Reservation volume

**Fig. 12:**
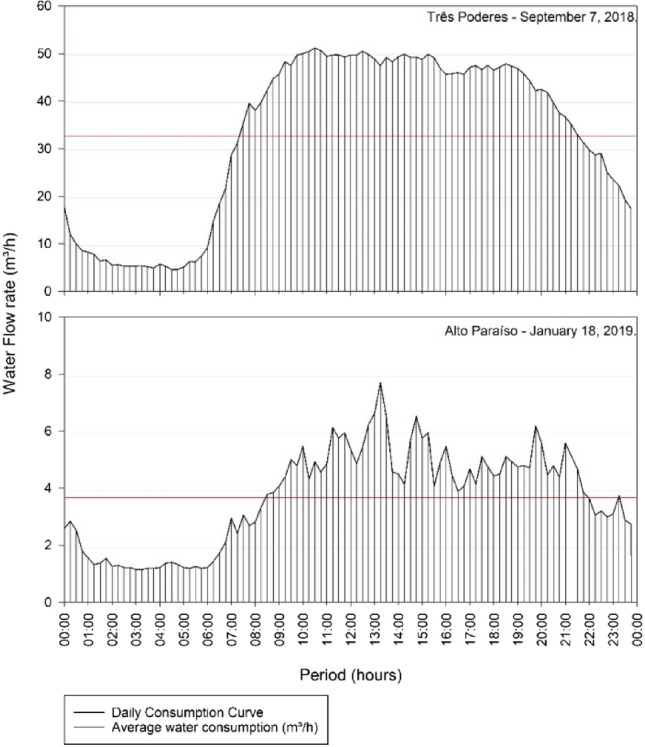
Analysis of the Consumption Curve of the Elevated Reservoirs of Itaguari

According to the current normative recommendations [15] and using the calculation parameters for the Reservation Volume equation defined in Eq. (11), parameters those, illustrated by the Consumption Curves of Fig. 12, the volume of the Reservoir Elevated Três Poderes and Alto Paraíso corresponds, respectively, to 182.89 m^3^ and 17.82 m^3^.

On the item 5.1.2 of the normative [15], referring to the specific conditions of the Volume of the reservoir, it is foreseen that the factor 1.2 is applied to the calculated volume, in order to consider any uncertainty in the analysed data. Therefore, the volume of the Três Poderes elevated reservoir calculated by the method of the effective normative [15] is 219.47 m^3^, that is, about 220 m^3^ and to the Reservoir Alto Paraíso approximately 21 m^3^.

Since the Sanitation Company of the state of Goiás uses the outdated normative [16] for the dimensioning of reservoirs, Table 12 provides the data used in order to determinate the two system’s volume of reservation.

**Table 12:** Project data to determine the reservation volume of Três Poderes and Alto Paraíso

System	No. of connections	Occupation rate	Consumption per capita	*k*_1_ coefficient
Três Poderes	1888	2.87	150	1.2
Alto Paraíso	204			

Therefore, according to the outdated normative [16], and through Eqs. (12) and (13), the volume of reservation for the Três Poderes system is determined as 325.15 m^3^, that is, about 325 m^3^, having the company adopted the maximum standard volume for an elevated reservoir of 200 m^3^ and complemented with a surface reservoir of 150 m^3^.

As for the Alto Paraíso system, the volume of the Alto Paraíso elevated reservoir calculated by the method of the outdated normative [16] is 35.28 m^3^, that is, about 35 m^3^. The company adopted the standard minimum volume for an elevated reservoir of 50 m^3^.

For both systems, Três Poderes and Alto Paraíso, there was a difference in the volume calculated when in the different prerogatives of the studied standards [15, 16], as illustrated in Table 13. It was observed that the outdate normative [16] provides an oversizing of approximately 68% for the Três Poderes and 60% for the Alto Paraíso reservoir, more than the necessary volume calculated by the actual normative [15].

**Table 13:** Comparison of volume by the two different normative

System	Method	Reservation Capacity (m^3^)	projected arrangement
Três Poderes	Outdated normative	325	1 Elevated reservoir of 200 m^3 ^+ 1 surface reservoir of 150 m^3^
	Actual normative	220	1 Elevated reservoir of 200 m^3^ + 1 superficial reservoir of 50 m^3^
Alto Paraíso	Outdated normative	35	1 Elevated reservoir of 50 m^3^
	Actual normative	21	

It is important to note that generally the total volume of reservation is considered taking into account the useful volume, the fire reserve and a reserve for emergencies represent by the useful volume plus the largest volume among the reserves for fire and emergencies [19]. In the Water Supply System adopted by the state company, these reserves are not considered in the size of the reserve volume, as fires occur relatively in a low frequency event in small- and medium-sized cities. Instead, hydrants connected to the network of distribution are still used, and the fire reserve is compulsorily used in residential reservoirs. If this criterion were considered to define the volume of the reservoir in the Water Treatment System, there would be a need for more robust reservoirs and consequently the cost of implantation would be increased considerably.

In addition, as one the essential aspects of the Brazilian water system, the water tanks have the main function of regularizing the water flow in the system. This elementary function supplies demand variations over the 24 h of the day, ensuring water supply for a peak flow event, accumulating water during periods of when the consumption is lower than average.

Although there is a difference in the calculation parameters used in each normative, what justifies the difference in the results obtained, it is important to note that both methods take into consideration the same system behaviour—abduction of water during 24 h for the day that presents the worst-case scenario of operation.

It is understood that, due to the interference of the system operator, the water tanks always operate in full, making it impossible to collect data in the flow metre to analyse its behaviour. In the case of the city of Itaguari, the modern and automated water system demands the least interference of the operator, maintaining the greatest data reliability among the small systems in the state, and providing conditions to the implement of the consume variation curve in the dimensioning of the water tanks.

**Fig. 13:**
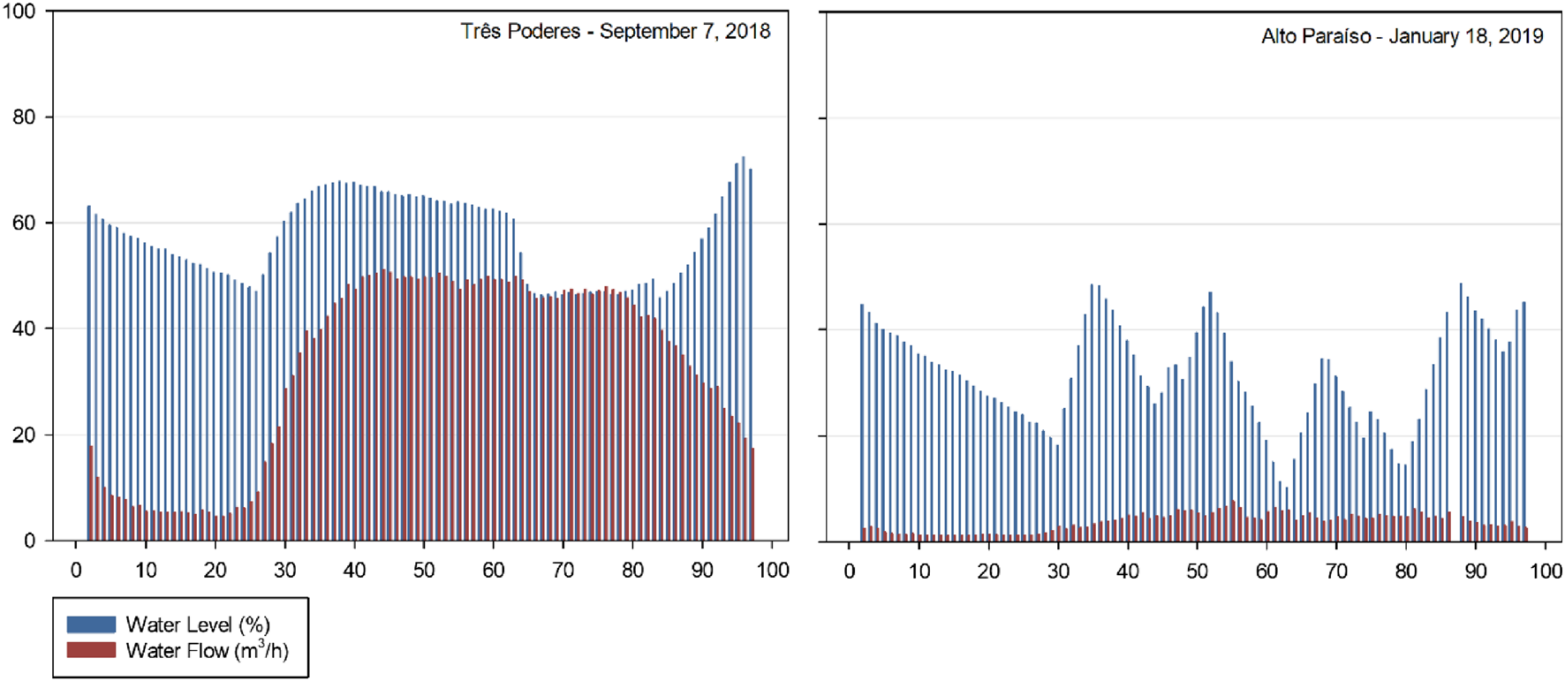
Reservation system behaviour—the water level inside the tanks in comparison to the consume while operating in extreme event

In Fig. 13, it is possible to observe the explained behaviour of the system in relation to the percentual level of water operating in the water tanks and the water consume in the reservation unit along the day, when operating in extreme events—day of highest consumption.

**Fig. 14:**
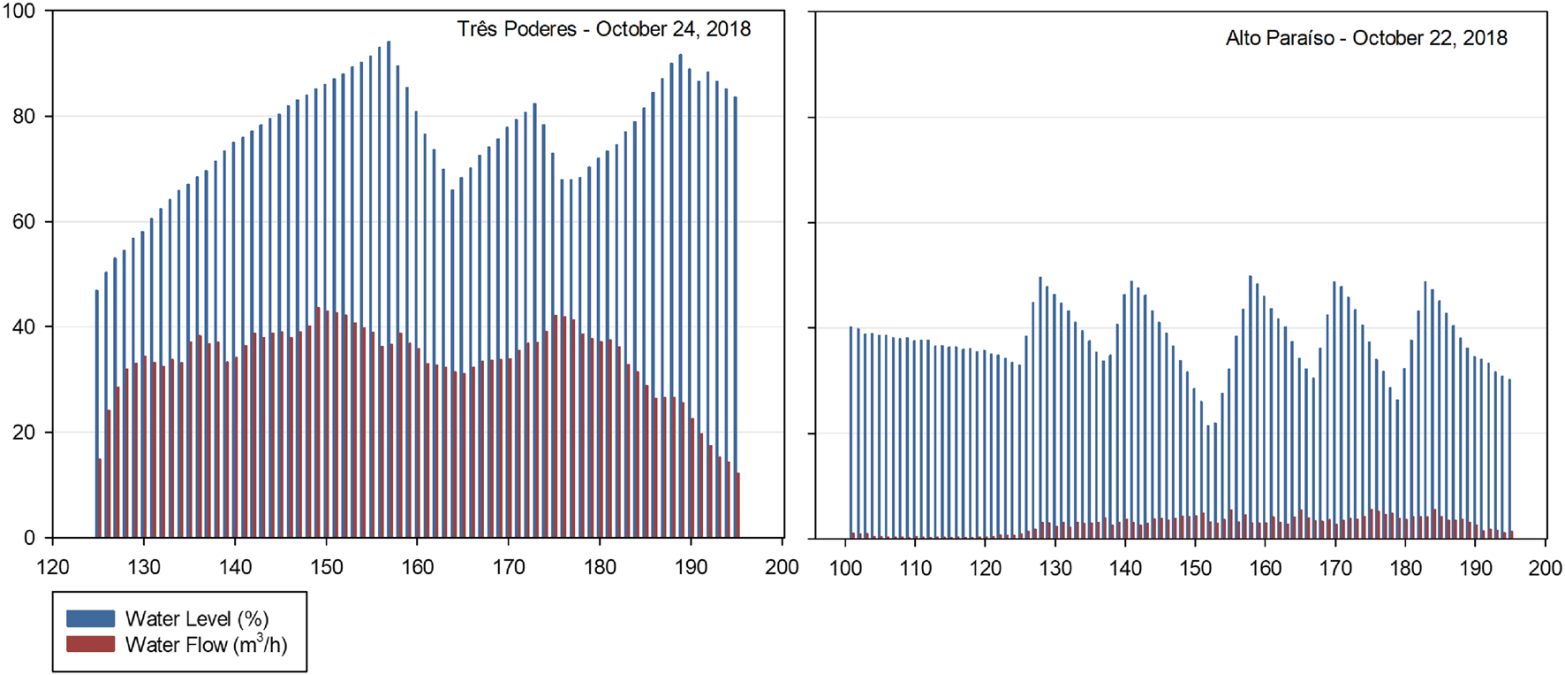
Reservation system behaviour—the water level inside the tanks in comparison to the consume while operating in conventional event

The same behaviour occurs while operating in function of the average consume—conventional event, characterized by the day that represents the average consume during the year, according to Fig. 14.

With the two elevated reservoirs remaining with the same capacity of 200m^3^ and 50m^3^, as proposed in Table 13, these units remain operating with the same behaviour and are still able to cope with the same conventional and pick flow events. When taking into consideration the consumption curve, in this case, the proposed change relies on the capacity of the surface reservoir that complements the Três Poderes reservation system, where the water tank volume is significantly smaller than the volume calculated by the old normative.

However, it is important to note that this study considerate the actual demand and was not applied considering a 20-year period project; therefore, in this scenario, the authors recommend the surface reservoir to remain with the actual capacity of 150 m^3^ in order to cope with future demand.

Regarding the Alto Paraíso reservoir, as the company adopts a minimum volume of 50 m^3^ for elevated reservoirs, the system would remain the same.

With these results the authors proposed a reducing when considering the actual demand, but factors such as the measurement data accuracy, the population growth and the climate changing over the years, prove we cannot rely on a single curve to represent the trend of highest consume. Statistically speaking, it is needed more experiments in order to apply a regression curve that fits a distribution of data, namely water flow measurements, that represent at best the consume of the population.

**Fig. 15:**
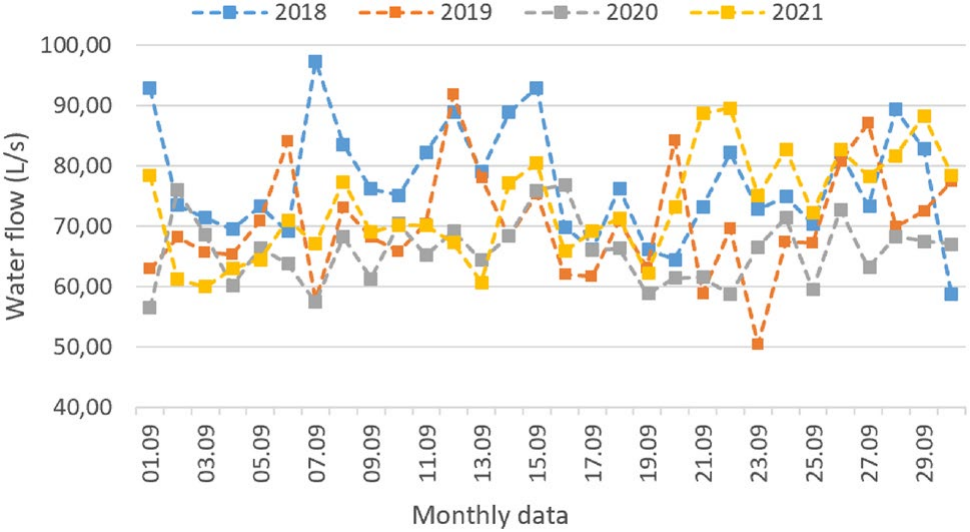
Generic model graphic data to exemplify the distribution of the water flow in the water tanks, during different years

The extreme curve that represents at best the high consumption curve includes wider range of data other than a year. With enough data, it is possible to plot the graphic distribution of the water flow for different years, as expressed in the generic model of Fig. 15 and, with this plot distribution, demonstrates how reliable is the consume curve for a long-term analysis.

After that, we can apply different linear regression models in order to determinate a consumption curve that fits best to represent the consume along the years.

Furthermore, the analysis must consider unpredictable events that changes the population consumption behaviour, as it occurred during the lockdowns due the Covid-19 pandemic. This event probably changed the behaviour of consumption.

In resume, even if the consumption variation curve predicts the oversizing of the reservation system in 68% and 60% for each unit, it does not mean that the system has to be reduced. This because a one-year analysis is only one part of the population behaviour and does not reflect a future system comportment.

The advantage of this analysis is that, increasing the data range to more than one year, adding climate aspects and the population growth tendency, we have enough data to get reliable results and analyse the real impact of the consumption curve in the dimensioning of the water tanks.

## Conclusion

1. High-rate filtering is required to meet acceptable Water Quality Index parameters for drinking water intended for human consumption, raising the operating capacity to 360 m^3^/ (m^2^.day).
2. This measure should be combined with the expansion of the Water Treatment Station units, as the station currently operates at the limit capacity of 33 L/s.
3. The use of the design criteria of the outdated normative, for dimensioning the reserve capacity, by the State Sanitation Companies and by area designers, generates a less precise analysis and therefore can generate an over-dimensioning in the volumes of the Reservoirs.
4. Even though the outdated normative provokes an oversized dimension of 68% and 60% for each reservoir, it does not mean that the system has to be reduced in capacity.
5. For future studies, it is recommended to analyse how the study of the consumption curve could be better used to determine the volume projections in the 20-year project period.
